# Validation and Psychometric Testing of the Chinese Version of the Mental Health Literacy Scale Among Nurses

**DOI:** 10.3389/fpsyg.2021.791883

**Published:** 2022-01-26

**Authors:** Anni Wang, Shoumei Jia, Zhongying Shi, Xiaomin Sun, Yuan Zhu, Miaoli Shen

**Affiliations:** ^1^School of Nursing, Fudan University, Shanghai, China; ^2^Shanghai Mental Health Centre, Shanghai, China; ^3^Shanghai Tongji Hospital, School of Medicine, Tongji University, Shanghai, China; ^4^Shanghai Chest Hospital, Shanghai Jiao Tong University, Shanghai, China; ^5^Changning District Mental Health Center, Shanghai, China

**Keywords:** mental health literacy, instrument development, validity, reliability, nurse, health professional

## Abstract

The Mental Health Literacy Scale (MHLS) is the most widely used and strong theory-based measurement tool to gain an understanding of mental health knowledge and ability. This study aimed to test the psychometric properties of the Chinese version of the Mental Health Literacy Scale (MHLS-C) and to document the norm and its influential factors of mental health literacy among nurses. The MHLS was translated following Brislin’s translation model and tested with a sample of 872 clinical registered nurses. The Jefferson Scale of Empathy-Health Professionals (JSE-HP), Patient Health Questionnaire-2 (PHQ-2), and Generalized Anxiety Disorder-2 (GAD-2) were administered to assess convergent validity. The minimum average partial test, parallel analysis and confirmatory factor analysis supported 4 first-order 2 second-order structure. The 4 factors were named “knowledge of mental disorder,” “ability to seek information and help,” “recognition of mental disorder,” and “acceptance of patients with mental illness,” with factor 1-3 were summarized into MHLS-Core (Core literacy subscale) and factor 4 as MHLS-SA (Social acceptance subscale). The MHLS-C was moderately negatively correlated with the PHQ-2 and GAD-2 (-0.111, -0.081) and highly positively correlated with JSE-HP (0.492). The Cronbach’s α was 0.85 for the overall scale and 0.89 and 0.93 for two subscales. The test-retest reliability was good, with intraclass correlation coefficients (ICCs) of 0.80 for the whole scale, and 0.79 and 0.94 for two subscales. As an approximately normal distribution, the 50*^th^* percentile for the MHLS-C was 99, with 50*^th^* percentiles of 74 and 20 for MHLS-Core and MHLS-SA. Higher position, higher professional credentials, higher hospital hierarchy, other specialist hospital, psychiatric hospital and unmarried status were positive predictors. The 29-item MHLS-C, with two subscales of MHLS-Core and MHLE-SA, is a stable and validated tool to measure mental health literacy. MHLS-Core could be used independently to measure the core content of mental health literacy. It may be applicable for Chinese health professionals, but need further validation among the general public. MHL curriculum and a targeted culturally appropriate program for acceptance for health professionals, especially for those in general hospitals and with less working tenure, may be recommended.

## Introduction

In the past three decades, mental disorders have become more prevalent and one of the top causes of diseases worldwide (World Health Organization at https://www.who.int/health-topics/mental-health#tab=tab_2). Calculated by years lived with disability (YLD), the disease burden of mental disorders exceeds 1/5 of the total global disease burden (Disease and Injury Incidence and Prevalence Collaborators [DIIPC], 2018). Mental health literacy (MHL) is a newly proposed construct that refers to knowledge and attitudes regarding mental health that aid in the recognition, management and prevention of mental health issues and has attracted much scholarly attention recently (Jorm et al., 1997). Studies have confirmed that MHL is an important factor affecting mental health (Jorm et al., 1997). Individuals with high MHL have more knowledge about mental health, hold less stigmatized attitudes toward mental illness, and more easily identify mental illness and adopt appropriate use of psychological service resources, whereas individuals with low MHL often adopt inappropriate or even incorrect coping (such as alcohol or other drugs), delay help-seeking behavior and prematurely abandon psychotherapy (Jorm et al., 1997; Rüsch et al., 2011; Kutcher et al., 2016). The general public’s MHL is generally low even in developed countries (Jorm et al., 1997; Jorm, 2012; Kutcher et al., 2016) and developing countries such as China (Jiang et al., 2020), and thus, enhancing MHL is of great significance. This issue has also been taken seriously by China Health Commission in its “Healthy China Action (2019–2030),” which took the improvement of mental health literacy as an important national strategic goal for improving the level of mental health of the people (accessible at: http://www.gov.cn/zhengce/2019-07/16/content_5410295.html).

According to Jorm et al., MHL consists of seven attributes: the ability to recognize specific disorders; knowing how to seek mental health information; knowledge of risk factors and causes; knowledge of self-treatments; knowledge of professional help available; and attitudes that promote recognition and appropriate help-seeking (Jorm et al., 1997). Based on this, valid scales that measure this construct and assess all attributes of MHL have recently been developed, including the Mental Health Literacy Scale (O’Connor et al., 2014), the Mental Health Literacy Measure (Jung et al., 2016), the Mental Health Literacy Questionnaire for young people (Dias et al., 2018), and Mental Health Literacy in Healthcare Students (Chao et al., 2020). Among those scales, the Mental Health Literacy Scale (MHLS) poses rigid Consensus based standards for the selection of health Instruments (COSMIN) methodological quality and can be applied to general people judged by its item content (Mokkink et al., 2010), whereas the other scales mentioned above were for public housing staff, young people, or healthcare students. Therefore, once developed, MHLS has been rapidly used, and until now, it has been validated in Persian (Heizomi et al., 2020) and Iranian (Nejatian et al., 2021) versions among the general population. Additionally, content validation by expert panels has been conducted for primary health care workers in South Africa and Zambia (Korhonen et al., 2019). This indicated that MHLS has the potential to be used among health professionals.

The mental health literacy of medical staff is very important, as it not only maintains their own mental health but also helps them understand the manifestation of patients and provides patients with better mental health care and humanistic care (Jorm et al., 1997). The few surveys that have been conducted have shown that the mental health literacy of health care practitioners or nurses is insufficient, and there is still a certain gap between the actual needs for dealing with their mental disorders (Elyamani and Hammoud, 2020; Hao et al., 2020). Before entering the intervention stage to promote MHL among health professionals, it is necessary to validate a tool to measure this parameter. However, the Chinese version of the MHLS was only initially validated among teachers with a small sample (Chen et al., 2021).

The existing versions of the MHLS suggest that a different structure and possible item redundancy may emerge in a Chinese context. Because MHLS were found to have six factors in the Iranian version, five factors in the Persian version, and one factor in Chinese version among teachers, and these different versions deleted different items which were highly culture and context sensitive (Heizomi et al., 2020; Chen et al., 2021; Nejatian et al., 2021). Therefore, the purpose of this study was to psychometrically test the Chinese version of the Mental Health Literacy Scale (MHLS-C) and to document the level and influential factors of MHL among nurses. This will provide the needed measurement tool of MHLS for the Chinese population and lay the foundation for validation studies for health professionals in other countries.

## Materials and Methods

We set up a translation group for formative translation work and cognitive interviews (Phase One), and then went to subsequent validation procedures (Phase Two).

### Translation

The MHLS-C was developed in two phases. Phase One was for item translation adapted from the MHLS (O’Connor and Casey, 2015). Permission to translate and validate the MHLS was obtained from Dr. O’Connor, the original designer. We translated the MHLS into the MHLS-C based on an adapted Brislin’s translation model for cross-cultural translation, which included translation, back-translation, comparison, linguistic adaptation and pilot testing (Brislin, 1970). First, two bilingual researchers independently translated the 35-item MHLS from English to Chinese and combined these two versions. Then, a third bilingual researcher back-translated the Chinese version into English. Later, a fourth researcher compared the back-translated English version with the original English scale. Disagreements were mainly derived from the different expression habits with respect to word order. A consensus was reached via a group discussion. This process yielded Chinese version 1 of the MHLS-C.

Individual cognitive interviews were conducted by the principal investigator with 20 clinical nurses. This method was used to how well participants understood the questions and could provide valid responses reflecting their own symptom experiences (Reeve et al., 2017). The interview used structured questions to detect how residents interpreted the items and thereby tested their comprehensibility and readability. Example questions include “Tell me in your own words what this question is asking” and “What does this item mean to you?” The information from the interviews was discussed in a group meeting, and we made appropriate changes to a few synonyms, such as by changing “紊乱” to “障碍” and “政客” to “政界人士.” This process yielded Chinese version 2 of the MHLS-C for validation.

### Participants

Phase Two tested the psychometric properties using a cross-sectional survey. The inclusion criteria were (1) registered nurses with a working experience over 1 year (usually finishing fresh nurse training), (2) aged 18–60 years old, and (3) voluntary participation. In April 2021, a stratified-clustered sample of participants was recruited. The researchers distributed the e-invitation and questionnaire link via the widely used smartphone-based investigation tool Wenjuanxing.^1^ Using stratified cluster sampling, we chose 15 out from 35 tertiary and 15 out from 54 secondary public hospitals in Shanghai, including comprehensive, psychiatric and other specialized hospitals. In each hospital, we randomly selected 2 to 3 wards. The link was disseminated by the head nurse in the selected wards, and all nursing staff who met the inclusion criteria were willing to participate in the survey.

Sample size was determined based on the subject-to-item ratio of 5–10:1 (Streiner et al., 2015) based on a set of 35 items. The sample size ranged from 350 to 700 for exploratory factor analysis (EFA) and confirmatory factor analysis (CFA), respectively. When the sample size neared the required number, the researcher ended the questionnaire. A total of 872 registered nurses were recruited. The sample was split into two parts for analysis as stated above. The characteristics of the nurses are shown in Table 1, and there was no significant difference between the two randomly selected groups in any of the sociodemographic or family characteristics. To evaluate the test-retest reliability, after 2 weeks, 40 participants in two wards were asked to complete the MHLS-C again.

**TABLE 1:** Socio-demographic and family characteristics of participants.

Characteristics		Total (*N* = 872)	Group 1 (*N* = 440)	Group 2 (*N* = 436)	χ^2^/t	P
		N (%)	N (%)	N (%)		
Gender	Male	28(3.2)	16(3.6)	12(2.8)	0.51	0.47
Marital status	Unmarried	273(31.3)	139(31.6)	134(31.0)	1.95	0.58
	Married/Remarried	577(66.2)	291(66.1)	286(66.2)		
	Divorced/Widowed	22(2.6)	10(2.3)	12(2.7)		
Educational level	Vocational education	40(4.6)	20(4.5)	20(4.6)	0.05	0.99
	College	302(34.6)	153(34.8)	149(34.5)		
	University	511(58.6)	257(58.4)	254(58.8)		
	Postgraduate	19(2.2)	10(2.3)	9(2.1)		
Type of hospital	Comprehensive	498(57.1)	260(59.1)	238(55.1)	3.04	0.21
	Other specialist	105(12.0)	45(10.2)	60(1.39)		
	Psychiatric	269(30.8)	135(30.7)	134(31.0)		
Hospital Hierarchy†	Tertiary	779(89.3)	394(89.5)	385(89.1)	0.27	0.87
	Non-tertiary	93(10.1)	46(10.5)	47(10.9)		
Position	Clinical nursing	720(82.6)	362(82.3)	358(82.9)	2.99	0.70
	Nurse Manager/Director/Chief	152(17.4)	78(17.7)	74(17.1)		
Professional credentials	Licensed Vocational Nurse	218(25.0)	120(27.3)	98(22.7)	2.62	0.62
	Registered Nurse	424(48.6)	208(47.3)	216(50.0)		
	Nurse Practitioner	201(23.1)	98(22.3)	103(23.8)		
	Advanced Practice Nurse	29(3.4)	14(3.2)	15(3.5)		
	Range	M (SD)	M (SD)	M (SD)		
Age (years)	20–58	35.23 ± 8.86	34.96 ± 9.07	35.49 ± 8.64	0.88	0.37
Clinical tenure (years)	1–38	14.18 ± 9.80	13.82 ± 9.89	14.54 ± 9.71	1.07	0.28

*^†^In China, health service institutions are rated into three levels: Primary, Secondary and Tertiary. Tertiary hospitals are large and gather the best medical resources.*

### Measures

A questionnaire consisting of several sociodemographic questions and several scales was used. Completion of the questionnaire required approximately 3–10 min. The JSE-HP, PHQ-2, and GAD-2 were applied to evaluate convergent validity.

#### Socio-Demographic Characteristics

The sociodemographic characteristics included sex, marital status, educational level, type of hospital, hospital hierarchy, position, professional credentials, age, and clinical tenure.

#### Mental Health Literacy Scale

This scale was developed by Matt O’Connor in 2015 (O’Connor and Casey, 2015) and is theoretically based on the concept of mental health literacy (Jorm et al., 1997). It contained 35 items, of which the first 15 items had a 4-point scaling (1–4), and the remaining 20 items had a 5-point scaling (1–5). The original scale contains only one dimension. The total score ranges from 35 to 160, with a higher score reflecting better mental health literacy. The Cronbach’s alpha coefficient (α) was 0.86 in this study.

#### Jefferson Scale of Empathy-Health Professionals

This is a mature scale developed to measure the empathy ability of health professionals (Samuel et al., 2007). This scale contains 20 items and uses a 7-point scaling; a higher score reflects better empathy capability. The Chinese version was validated with good validity and reliability (Hsiao et al., 2012). The Cronbach’s alpha coefficient (α) was 0.86 in this study.

#### Patient Health Questionnaire-2

It is a validated and short tool to measure depression and has been validated among the Chinese population (Yu et al., 2011). The total score ranges from 0 to 6, with a cut-off of 3. The Cronbach’s alpha coefficient (α) was 0.83 in this study.

#### Generalized Anxiety Disorder-2

It is a validated and short tool to measure anxiety and has been among the Chinese population (Kroenke et al., 2009). The total score ranges from 0 to 6, with a cut-off of 3. The Cronbach’s alpha coefficient (α) was 0.87 in this study.

### Ethical Consideration

The study was reviewed by the Institutional Review Board at the researchers’ institute, and informed consent was obtained from all participants. The survey was anonymized. Data were downloaded, and only the researchers had access to them.

### Statistical Analysis

**FIGURE 1:**
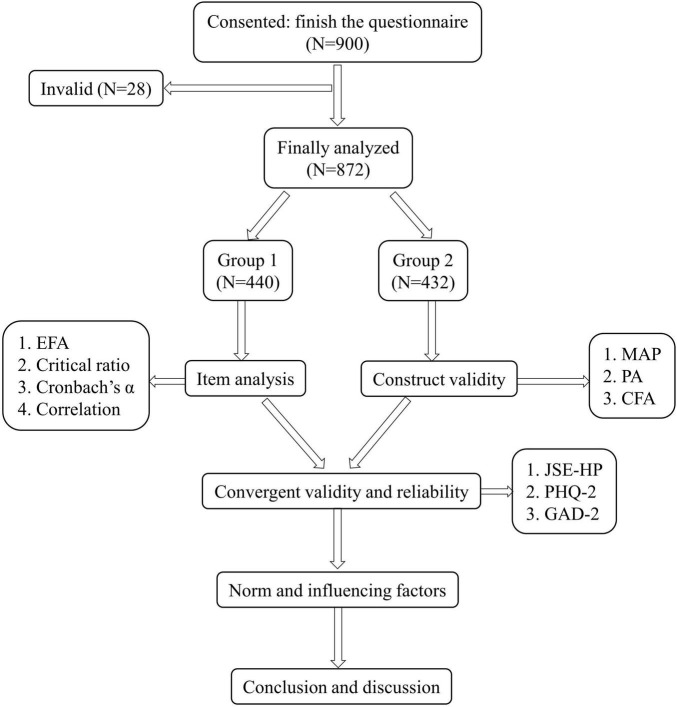
The process of the validation of MHLS-C.

The data were analyzed using SPSS 21.0 (IBM Corp. Released 2010. Armonk, NY: IBM Corp.) and Mplus 7.0 (Muthén and Muthén). The methodological quality of the MHLS was examined using the COSMIN checklist (COnsensus-based Standards for the selection of health status Measurement INstruments) (Mokkink et al., 2010). In total, six of the nine domains were determined to be adequately assessed: item analysis, internal consistency, retest reliability, concurrent validity, structural validity, and hypothesis testing. The sample of nurses was randomly split into two parts for EFA and CFA by using “select cases” function in SPSS, which meant 50 percentages of the total sample was randomly selected for EFA and the left was for CFA. The validation procedure is shown in Figure 1.

(a) Item analysis: We deleted an item if it met one of following criteria: (1) factor loading < 0.4 or cross-loading or false loading which could not be theoretically explained via EFA, (2) critical ratio (CR) value was non-significant with insufficient power of discrimination (Nunnally and Bernstein, 1994), (3) Cronbach’s α coefficient of the scale increased if this item was deleted (Nguyen et al., 2014; Huang et al., 2017), and (4) Pearson correlation coefficient between a certain item with total score was very small or non-significant, which reflected its representativeness and common variance with the whole scale is poor in explain that the connotation of the measurement (Nunnally and Bernstein, 1994).

In EFA analysis for detecting the number of factors and item loadings, we first tested whether the KMO value was >0.7 and whether the P value of Bartlett’s/df was <0.05, indicating suitability for factor analysis. Then, principal component analysis with the maximum variance method was conducted, and the solutions were examined theoretically (Floyd and Widaman, 1995). The number of extracted factors was based on an eigenvalue >1.0 and factor loading above 0.4 and the percentage of the explained variance.

(b) Structural validity: The EFA-derived structure was investigated using Velicer’s minimum average partial (MAP) test and a parallel analysis (PA) for a retest (O’Connor, 2000). According to Velicer, the smallest average squared partial correlation or the smallest average 4th-power partial correlation best indicates the number of factors (Velicer et al., 2000). One hundred random datasets were generated, and when the eigenvalue of the actual data became smaller than the corresponding eigenvalue of the random data, the number of factors was retained (O’Connor, 2000).

CFA was then conducted, and the parameters used to appraise the model were χ^2^/df (> 5) and several model fit indicators, including comparative fit index (CFI), Tucker–Lewis index (TLI), the Akaike information criterion (AIC), Bayesian information criterion (BIC) and root mean square error of approximation (RMSEA) (Kline, 2011). Comparative fit index and TLI values > 0.90 and RMSEA values < 0.08 suggest good fit, while the value for the AIC and BIC should be smaller to obtain the most parsimonious model fit (Kline, 2011). The first 15 items of the MHLS had a 4-point scaling, with the others were 5-point scaling. Thus, the robust weighted least squares with mean and variance adjustment (WLSMV) estimator was used in present study (Flora and Curran, 2004; Wang et al., 2013).

(c) Concurrent validity: Pearson correlation analysis was used to detect the relation of the total score and each factor of the MHLS-C with the JSE-HP, PHQ-2, and GAD-2. We anticipated that the mental health literacy score would have a positive relationship with empathy ability Jefferson Scale of Empathy-Health Professionals (JSE-HP) and a negative relationship with depression (PHQ-2) and anxiety (GAD-2).

(d) Reliability: Cronbach’s α coefficient was applied to determine the internal consistency of the whole scale and its dimensions. The intraclass correlation coefficient (ICC) was used to detect the correlation in 40 nurses at a two-week interval.

(e) Statistical description was used to show the norm, and regression analysis was performed to detect the variables influencing mental health literacy. For unranked variables, such as marital status and working hospital, dummy variables were created.

## Results

### Item Analysis of Mental Health Literacy Scale: Exploratory Factor Analysis, Critical Ratio, Cronbach’s α, r Value

The KMO value was 0.92, and Bartlett’s test of sphericity was significant (chi square = 21613.87, *P* < 0.001). Then, in EFA analysis, four factors were extracted that accounted for 66.33% of the variance. The loading coefficient from EFA, CR values, Cronbach’s α coefficient after deleting an item, and r values of each item are summarized in Table 2. According to the criteria for item retention. I-10, I-15, I-20, I-21, I-22, and I-23 were deleted for false loading not complied with theoretical understanding, cross-loading, the CR value was non-significant, and the correlation coefficient was low.

**TABLE 2:** Item analysis of MHLS-C.

	EFA					Critical ratio (CR)	Cronbach’s α after removing this item†	r value with total score	Item retention
Item	Factor1	Factor2	Factor3	Factor 4	Factor 5				
1	0.47					6.602	0.82	0.38**	
2	0.70					12.089	0.81	0.49**	
3	0.72					13.578	0.81	0.52**	
4	0.75					13.987	0.81	0.50**	
5	0.80					15.438	0.81	0.54**	
6	0.82					16.304	0.81	0.57**	
7	0.82					16.876	0.81	0.59**	
8	0.79					15.084	0.81	0.55**	
9	0.60					8.791	0.81	0.37**	
10	0.40		0.42			2.023	0.83	0.08**	No
11	0.74					12.967	0.81	0.48**	
12	0.52					5.157	0.83	0.18**	
13	0.74					14.513	0.81	0.52**	
14	0.56					11.385	0.81	0.40**	
15		0.47	0.42			1.817	0.81	0.08*	No
16		0.60				13.400	0.82	0.42**	
17		0.60				10.023	0.81	0.35**	
18		0.61				13.400	0.81	0.45**	
19		0.66				11.731	0.82	0.42**	
20		0.51		0.34		2.528	0.83	0.08*	No
21		0.44	0.43			9.455	0.81	0.37**	No
22	0.48					11.207	0.81	0.43**	No
23		0.49	0.40			5.488	0.81	0.26**	No
24				0.59		18.030	0.81	0.54**	
25				0.62		15.189	0.81	0.48**	
26				0.61		17.636	0.82	0.52**	
27				0.60		18.386	0.81	0.52**	
28				0.61		18.613	0.81	0.51**	
29					0.76	8.968	0.81	0.35**	
30					0.79	12.904	0.81	0.44**	
31					0.79	12.962	0.81	0.45**	
32					0.80	11.361	0.81	0.41**	
33					0.72	3.189	0.83	0.09*	
34					0.70	9.478	0.81	0.33**	
35					0.76	9.469	0.81	0.33**	

*P < 0.05, **P < 0.01; ^†^The overall Cronbach’s α of 32 items = 0.82.

### Construct Validity of Mental Health Literacy Scale: Minimum Average Partial, Parallel Analysis and Confirmatory Factor Analysis

In the MAP test, when the root was 4, we obtained the smallest average squared partial correlation and the average 4th-power partial correlation get larger in root 5. In the parallel analysis, when the root was 5, the mean random-data eigenvalue was larger than its eigenvalue from the actual dataset. Therefore, based on the MAP test and PA shown in Table 3, a 4-factor model is recommended and statistically stable.

**TABLE 3:** MAP and PA test of MHLS-C.

Root	MAP test		PA	
	Average part r sq	Average part r 4th	Raw data	Means
0	0.0.89	0.0394	-	-
1	0.0603	0.0199	8.0580	1.3454
2	0.0426	0.0075	5.4673	1.2987
3	0.0356	0.0053	3.3936	1.2633
4	0.0147	0.0107	2.4752	1.2327
5	0.0157	0.0129	1.0344	1.2051
6	0.0158	0.0176	0.8823	1.1794
7	0.0187	0.0268	0.7071	1.1553

*MHLS-C = Mental Health Literacy Scale-Chinese.*

The 4-factor solution was then validated using the CFA sample. The CFA confirmed the 4-factor structure. To improve the model fit based on modification indices, several pairs of residual correlations within one factor were added, indicating that each element of a pair had the same facet as its attributed factor. As factor 4 was correlated with other factors with weak even negative correlation, we further detected the higher order structure. The fit indicators were summarized in Table 4. The 1 factor model based on original scale showed unsatisfactory model fit, and the 4 factor model as well as 4 first-order 1 second-order fit much better. Further, we detected 4 first-order 2 second-order model, of which factor 1, factor 2 and factor3 were summarized together while factor 4 was set alone. This 4 first-order 2 second-order structure showed equal indicators with 4 first-order 1 second-order. Thus, based on theoretical analysis, the 4 first-order 2 second-order structure is recommended.

**TABLE 4:** Fit indicators of each tested model.

Model	χ2	df	χ2/df	CFI	TLI	RMSEA	AIC	BIC
1 factor	10474.79	396	26.44	0.48	0.42	0.175	55489.84	55955.37
4 factor	1405.56	361	3.89	0.95	0.94	0.057	46432.31	46926.96
4 first-order 1 second-order	1472.42	363	4.05	0.94	0.94	0.058	46495.16	46980.21
4 first-order 2 second-order	1472.42	363	4.05	0.94	0.94	0.058	46495.16	46980.21

**FIGURE 2:**
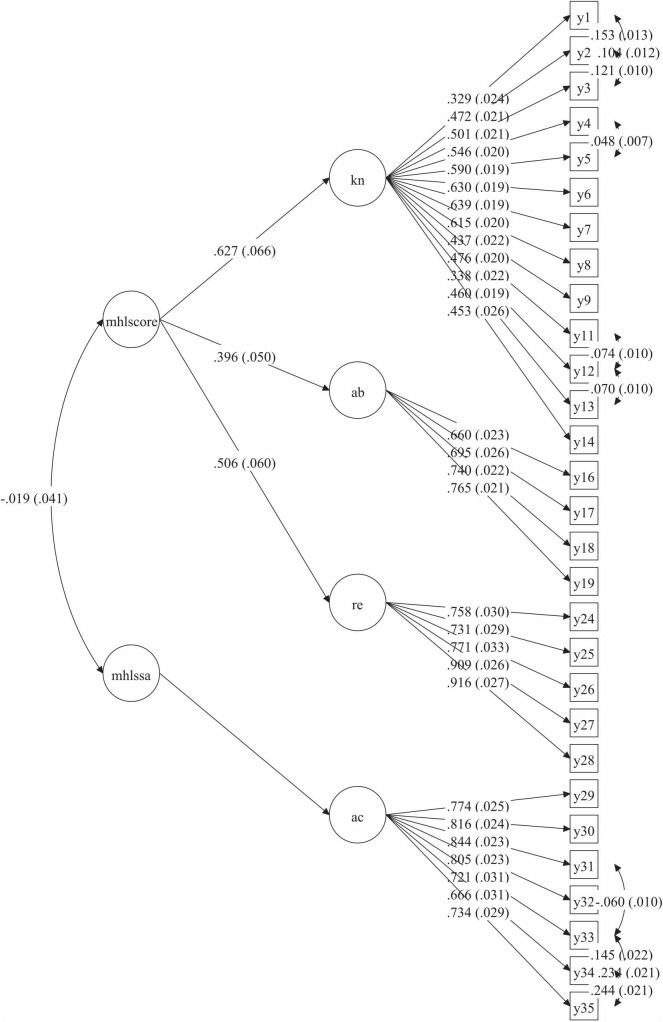
Final structure of MHLS-C*. *MHLS-C = Mental Health Literacy Scale-Chinese, mhlscore = Core literacy subscale, mhlssa = Social acceptance subscale, kn = Knowledge, ab = Ability, re = Recognition, ac = Acceptance.

The final structure is shown in Figure 2, with regression weights that were significant. Following theoretical analysis, we named factor 1 “knowledge of mental disorder (knowledge),” factor 2 “ability of seeking information and help (ability),” factor 3 “recognition of mental disorder (recognition),” and factor 4 “acceptance of patients with mental illness (acceptance).” The factor 1, factor 2 and factor 3 contributed to a higher latent variable defined as “Core literacy,” while factor 4 contributed to “Social acceptance,” which could be seen as two subscales, MHLS-Core and MHLS-SA.

### Concurrent Validity and Reliability of Mental Health Literacy Scale

Pearson correlation analysis showed that MHLS-C and MHLS-Core were moderately negatively correlated with the PHQ-2 and GAD-2 and highly positively correlated with the JSE-HP. While MHLS-SA was not significantly correlated with PHQ-2, GAD-2, JES-HP, and MHLS-Core but with total score. More specifically, MHLS-SA and knowledge were not significantly correlated with PHQ-2 and GAD-2 scores. All factors were significantly correlated with JES-HP, except MHLS-SA. More information could be found at Table 5.

**TABLE 5:** Correlation among MHLS-C, depression, anxiety and empathy.

	MHLS-C	MHLS-Core	MHLS-SA	Knowledge	Ability	Recognition	PHQ-2	GAD-2
MHLS-C	1							
MHLS-Core	0.847**	1						
MHLS-SA	0.476**	-0.021	1					
Knowledge	0.674**	0.791**	-0.044	1				
Ability	0.523**	0.500**	0.157**	0.206**	1			
Recognition	0.541**	0.676**	-0.103**	0.228**	0.207**	1		
PHQ-2	-0.111**	-0.128**	-0.005	0.043	-0.237**	-0.213**	1	
GAD-2	-0.081*	-0.100**	0.012	0.072	-0.215**	-0.196**	0.757**	1
JSE-HP	0.492**	0.605**	-0.019	0.363**	0.343**	0.534**	-0.248**	-0.200**

*MHLS-C = Mental Health Literacy Scale-Chinese, MHLS-SA = = Mental Health Literacy Scale-Social acceptance subscale, MHLS-Core = Mental Health Literacy Scale-Core Literacy subscale, PHQ = Patient Health Questionnaire-2, GAD-2 = Generalized Anxiety Disorder-2, JSEHP = Jefferson Scale of Empathy-Health Professionals.*

*P < 0.05 and **P < 0.01.

The Cronbach’s α was 0.85 for the overall scale, with 0.89 and 0.93 for two subscales. The test-retest reliability was good, with ICCs of 0.80 for the whole scale and 0.79 and 0.94 for two subscales.

### Descriptive Analysis of the Mental Health Literacy Scale and Known Group Test

The mean scores was 93.02 ± 10.76, The basic statistics and percentiles of each subscale are summarized in Table 6. The skewness and kurtosis of each factor and whole scale indicated that they were not strictly normally distributed. Thus, scores in each quartile of each factor and whole scale were used to describe the cut-off, as shown in Table 6. The 50*^th^* percentile for the MHLS-C was 99, with 74 and 20 for MHLS-Core and MHLS-SA.

**TABLE 6:** Descriptive analysis and percentiles of MHLS-C.

	Knowledge	Ability	Recognition	MHLS-SA	MHLS-Core	MHLS-C
Minimum	20	4	5	7	44	53
Maximum	51	20	25	35	96	127
Mean ± SD	41.18 ± 5.59	14.88 ± 2.93	17.58 ± 3.99	19.68 ± 5.30	73.63 ± 9.10	93.02 ± 10.76
Skewness	-0.85	-0.06	-0.28	0.05	-0.04	0.07
Kurtosis	1.20	-0.08	0.18	0.40	-0.20	0.27
Percentile						
95	49	20	25	28	89	111
**75**	**46**	**16**	**20**	**22**	**80**	**100**
**50**	**41**	**15**	**18**	**20**	**74**	**92**
**25**	**38**	**12**	**15**	**16**	**67**	**86**
5	28	11	10	11	60	75

*MHLS-C = Mental Health Literacy Scale-Chinese, MHLS-SA = = Mental Health Literacy Scale-Social acceptance subscale, MHLS-Core = Mental Health Literacy Scale-Core Literacy. Bold values represents percentile of each factor or whole scale.*

Setting the total MHLS-C score as the dependent variable, the regression analysis showed that professional credentials (β = 0.26, *P* < 0.001), hospital hierarchy (β = 0.13, *P* < 0.001), position (β = 0.12, *P* < 0.001), other specialist hospital (β = 0.10, *P* = 0.009), psychiatric hospital (β = 0.19, *P* < 0.001), entered into the model. The other sociodemographic variables had no statistical significance.

## Discussion

The present study is the first examination of MHLS among health professionals in China. The translation process was strictly conducted to ensure equivalence, and psychometric testing of the MHLS-C showed that the 29-item version with a two subscale structure is a reliable and valid tool for measuring mental health literacy.

### Validity, Reliability, and Application of Mental Health Literacy Scale

Robust psychometric characteristics and practicality are the basis for the clinical application of a tool. At the item level, three items were deleted for poor item information: I-10 “men are more likely to experience an anxiety disorder compared to women,” I-15 “a mental health professional can break confidentiality if your problem is not life-threatening and they want to assist others to better support you,” I-20 “People with a mental illness could snap out if it if they wanted,” I-21 “A mental illness is a sign of personal weakness,” I-22 “A mental illness is not a real medical illness” and I-23 “People with a mental illness are dangerous.” In the Iranian version, six questions (20, 21, 22, 26, 27, 28) were deleted by CFA (Nejatian et al., 2021), while in the Persian version, five items (10, 12, 20, 21, 22) were deleted from item analysis (Heizomi et al., 2020). We could see that I-10, I-20, I-21, and I-22 generally had poor item information in various versions. I-10 refers to gender risk factors for anxiety disorder, but the incidence between genders may be different in different countries, and thus it was deleted. I-20 refers to recognition of mental disorders, but it seems to be too absolute because mental illness could be self-cured in some cases, such as self-practiced mindfulness therapy (Parsons et al., 2017), and I-21, I-22 conveyed vogue content in Chinese context, and thus they had poor performance. I-15 did not perform well only in MHL-C, which may be due to cultural differences in China, where family members of patients with mental disorders highly participate in shared decision-making regarding admission, treatment, and discharge compared to European and Chinese Americans (Gao et al., 2019). I-23 was summarized to acceptance in Persian version and in our study, it was cross-loaded to ability to seeking help, which could not be explained theoretically. Therefore, 6 items deleted in the Chinese version were statistically, theoretically, and culturally appropriate.

Henson and Robert suggest that when performing EFA, the number of factors extracted should be considered based on multiple criteria rather than relying on a single standard alone (Reise et al., 2000; Henson and Roberts, 2006). The five-factor structure in MHLS-C was validated by EFA, CFA, MAP and PA. O’Connor’s and Jorm’s concept of MHL included several attributes, as mentioned in the introduction (Jorm et al., 1997; O’Connor and Casey, 2015). In the original English version of MHLS, as the communalities were low, the structure was univariate (O’Connor and Casey, 2015). In the Iranian version, six factors were the ability to recognize disorders, knowledge of self-treatment, knowledge of professional help available, knowledge of risk factors and causes, knowledge of where to seek information, and attitudes that promote recognition or appropriate help-seeking behavior (Nejatian et al., 2021). In the Persian version, five factors were the ability to recognize mental disorders, confidentiality of mental health practitioners, skills of mental health information seeking, beliefs about mental illnesses, and attitudes toward patients with mental illness (Heizomi et al., 2020). Compared with them, 4 factors in MHLS-C are more similar to the Persian version. Factor 1, “knowledge of mental disorder (knowledge),” refers to the ability to recognize specific disorders and the confidentiality of mental health practitioners in the Persian version (Heizomi et al., 2020) and all knowledge-related factors in the Iranian version (Nejatian et al., 2021). Factor 2 “ability to seek information and help (ability),” factor 3 “recognition of mental disorder (recognition)” and factor 4 “acceptance of patients with mental illness (acceptance)” were similar to the remaining factors in the Persian version accordingly (Heizomi et al., 2020). We could see the homogeneity and heterogeneity of structure in different cultures.

To be noted, acceptance with knowledge and recognition were either non-significant or negative, which was similar to the Persian version (Heizomi et al., 2020). The finding in this study complied with the findings in Persian version and also the definition of mental health literacy, which refers to knowledge and attitudes regarding mental health that aid in the recognition, management and prevention of mental health issues (Jorm et al., 1997). The criterion validity of MHLS-SA scores is poor both in our study and in Persian version, but good in the Iranian version. Further to compare the participants, the Persian version was among mothers of female high school children, whereas the Iranian version was among general population. Previous study has found that Chinese primary healthcare providers hold negative attitudes to mental health patients, especially with regard to engaging in closer personal relationships with psychiatric patients (Ma et al., 2018; Yin et al., 2020). The poor criterion validity of MHLS-SA may due to the testing participants, which may hold different level of acceptance and influence the correlation between acceptance and other factors. This implied that further validation of Chinese version among the general public may be necessary. In addition, this implies promoting knowledge and help-seeking, and an objective view does not guarantee people’s social acceptance of patients with mental disorders. When designing interventions, this attribute should be given independent attention. We further detected the higher 4 first-order 2 second-order structure, which showed that MHLS-C could be split into two subscales, core literacy and social acceptance. Even though criterion validity of MHLS-SA scores is poor, we recommended to keep it for future validation among other population group. The former core literacy subscale could be considered to be used independently to measure the core content of mental health literacy.

The concurrent validity of the MHLS-C was supported by its correlation with mental health status and empathy. In a previous study, there was no significant relationship between MHL and psychological distress (O’Connor and Casey, 2015), while other studies found similar results among university nursing students (Al-Yateem et al., 2018) and teachers (Chen et al., 2021). Our study found a moderately negative correlation with depression and anxiety, which implied that higher MHL would promote self-management and help-seeking and thus help to promote mental health status to some extent (Jorm, 2012). In addition, we found that MHL was highly positively correlated with empathy (0.586), which indicated that the promotion of MHL among health professionals would greatly promote their ability to observe and identify mental disorders and thus provide humanistic care ability. The Cronbach’s α coefficients and retest reliability of the whole scale and each factor were good, which indicated that the MHLS-C had good reliability.

### Level and Characters of Mental Health Literacy Among Nurses

As different versions contained different items, we used the ratio of the total score and mean score to compare the MHL level, which were 71.2% in our study, 71.5% in the Iranian public (Nejatian et al., 2021), 60.9% in Persian/Farsi speaking people (Heizomi et al., 2020), 72.0% in Chinese teachers (Chen et al., 2021) and 90.9% in Australian health professionals (O’Connor and Casey, 2015). We could see that the ratio in this study was moderately high but lower than that in Australian health professionals. A previous survey revealed that less than 50% of student nurses (Al-Yateem et al., 2018) and 54.3% of pediatric hospital staff in United Arab Emirates (Al-Yateem et al., 2017) could correctly identify the disorders presented and 38.9%, 56.2, and 17.5% for schizophrenia, depression, and GAD, respectively, in Chinese nurses in general hospitals (Hao et al., 2020). The results found that higher position, higher professional credentials, higher hospital hierarchy, other specialist hospital, psychiatric hospital and unmarried status were positive predictors of MHL. The results were in accordance with previous studies (Poon et al., 2019; Hao et al., 2020).

## Implications for Practice

The validated MHLS-C could facilitate assessment for nurses in clinical such as mental health evaluation, and/or non-clinical settings such as professional education training, and it has potential utility in other health care professionals although this requires further investigation and validation. Because its items reflected shared knowledge, attitude, recognition and attitude within health care professionals, not specific to a certain health profession. It may need further validation among the general public. The scale could be split to two subscales, Core Literacy and Acceptance, with the former one could be used independently to measure the core content of mental health literacy. The average score of this study revealed the need for an MHL curriculum and a targeted culturally appropriate program for acceptance for health professionals, especially for those in general and low hierarchy hospitals, and with low position and professional credentials.

## Limitations and Future Study

Although rigorous theoretical analysis and robust statistical analysis were applied, several limitations of this study should be considered. First, using a sample of registered nurses, we did not include doctors or other health-related professionals. Second, although stratified cluster sampling of a relatively large sample was used, the recommended reference score was based on nurses. Future studies could consider further applying it to multidisciplinary health professionals for further validation. Moreover, future studies could focus on the interaction of the factors within MHL, which will lay the foundation for developing programs to promote MHL.

## Conclusion

The psychometric properties found in this study indicated that the 29-item MHLS-C, with two subscales of MHLS-Core and MHLE-SA, is a stable, reliable and validated tool to measure mental health literacy. Mental health literacy was moderately negatively correlated with depression and anxiety and highly positively correlated with empathy. The recommended reference scores for MHLS-C, MHLS-Core and MHLS-SA were 99, 74 and 20, respectively. The MHLS-C is applicable to Chinese nurses and has potential utility in other health care professionals, but requires further investigation and validation among the general public. MHL curriculum and a targeted culturally appropriate program for acceptance for health professionals, especially for those in general and low hierarchy hospitals, and with low position and professional credentials.

## Data Availability Statement

The raw data supporting the conclusions of this article will be made available by the authors, without undue reservation.

## Ethics Statement

The studies involving human participants were reviewed and approved by Institutional Review Board of School of Nursing, Fudan University. The patients/participants provided their written informed consent to participate in this study.

## Author Contributions

AW: design, translation, and writing and data analysis. SJ: design, translation, data collection, and writing. ZS, XS, YZ, and MS: design, translation, and data collection. All authors listed meet the authorship criteria according to the latest guidelines of the International Committee of Medical Journal Editors and are in agreement with the manuscript.

## Conflict of Interest

The authors declare that the research was conducted in the absence of any commercial or financial relationships that could be construed as a potential conflict of interest.

## Publisher’s Note

All claims expressed in this article are solely those of the authors and do not necessarily represent those of their affiliated organizations, or those of the publisher, the editors and the reviewers. Any product that may be evaluated in this article, or claim that may be made by its manufacturer, is not guaranteed or endorsed by the publisher.

## Footnotes

1
http://www.wjx.cn
